# Serum Afamin a Novel Marker of Increased Hepatic Lipid Content

**DOI:** 10.3389/fendo.2021.670425

**Published:** 2021-09-16

**Authors:** Timea Kurdiova, Miroslav Balaz, Zuzana Kovanicova, Erika Zemkova, Martin Kuzma, Vitazoslav Belan, Juraj Payer, Daniela Gasperikova, Hans Dieplinger, Barbara Ukropcova, Jozef Ukropec

**Affiliations:** ^1^Department of Metabolic Disease Research, Biomedical Research Center, Institute of Experimental Endocrinology, Slovak Academy of Sciences, Bratislava, Slovakia; ^2^Department of Biological and Medical Sciences, Faculty of Physical Education and Sports, Comenius University, Bratislava, Slovakia; ^3^5^th^ Department of Internal Medicine, Faculty of Medicine, Comenius University, Bratislava, Slovakia; ^4^MRI Unit, Pro Diagnostic Group, Bratislava, Slovakia; ^5^Department of Genetics and Pharmacology, Institute of Genetic Epidemiology, Medical University of Innsbruck, Innsbruck, Austria; ^6^Department of Clinical Pathophysiology, Faculty of Medicine, Institute of Pathophysiology, Comenius University, Bratislava, Slovakia

**Keywords:** afamin, hepatic lipids, insulin resistance, prediabetes, type 2 diabetes, exercise

## Abstract

**Aim:**

Afamin is a liver-produced glycoprotein, a potential early marker of metabolic syndrome. Here we investigated regulation of afamin in a course of the metabolic disease development and in response to 3-month exercise intervention.

**Methods:**

We measured whole-body insulin sensitivity (euglycemic hyperinsulinemic clamp), glucose tolerance, abdominal adiposity, hepatic lipid content (magnetic resonance imaging/spectroscopy), habitual physical activity (accelerometers) and serum afamin (enzyme-linked immunosorbent assay) in 71 middle-aged men with obesity, prediabetes and newly diagnosed type 2 diabetes. Effects of 3-month exercise were investigated in 22 overweight-to-obese middle-aged individuals (16M/6F).

**Results:**

Prediabetes and type 2 diabetes, but not obesity, were associated with increased serum afamin (p<0.001). Afamin correlated positively with hepatic lipids, fatty liver index and liver damage markers; with parameters of adiposity (waist circumference, %body fat, adipocyte diameter) and insulin resistance (fasting insulin, C-peptide, HOMA-IR; p<0.001 all). Moreover, afamin negatively correlated with whole-body insulin sensitivity (M-value/Insulin, p<0.001). Hepatic lipids and fasting insulinemia were the most important predictors of serum afamin, explaining >63% of its variability. Exercise-related changes in afamin were paralleled by reciprocal changes in insulinemia, insulin resistance and visceral adiposity. No significant change in hepatic lipid content was observed.

**Conclusions:**

Subjects with prediabetes and type 2 diabetes had the highest serum afamin levels. Afamin was more tightly related to hepatic lipid accumulation, liver damage and insulin resistance than to obesity.

## 1 Introduction

The glycoprotein afamin is a member of the albumin gene family (1), expressed mainly in the liver (2), and secreted into the bloodstream, where it circulates at relatively high concentrations (3). Knowledge on the physiological role of afamin is limited. It was shown that afamin could serve as an extracellular vitamin E carrier (3, 4) and evidence exists that afamin could play an important role in neuroprotection (5), fertility (3), vitamin E bioavailability including its transport *via* the blood-brain barrier (6) as well as in bone metabolism/remodeling (7). Overexpressing afamin in mice increased body weight and serum cholesterol, triglyceride and glucose concentrations (8), indicating the role of afamin in systemic energy and glucose metabolism.

Afamin has been proposed to serve as an excellent clinically relevant marker of metabolic disease in the general population (8). Meta-analysis of large population studies clearly showed that plasma concentrations of afamin are strongly associated with prevalence and development of metabolic syndrome (8). A pooled analysis in more than 20.000 individuals confirmed that afamin was strongly associated with insulin resistance and that it could be considered an independent strong predictor of type 2 diabetes (T2D) (9). Each increase of afamin by 10 mg/L increases the probability of metabolic syndrome by 79% (8) and afamin predicts higher incidence of T2D independently from the other major metabolic risk factors (9). Increased afamin concentrations were identified as an early biomarker for gestational diabetes (10–12) and pre-eclampsia (13, 14) and have been positively associated with insulin resistance in patients with polycystic ovary syndrome (15, 16). Afamin is rather stable, it can be measured in serum or plasma, and its circulating values are not modulated by age, gender, menstrual cycle, prandial state or circadian rhythms (17). Taken together, afamin represents an excellent candidate biomarker for increased risk of metabolic disease.

The main source of circulating afamin is the liver (2). Excessive hepatic lipid accumulation is associated with non-alcoholic fatty liver disease, hepatic insulin resistance and, increased risk of T2D. However, severe alcoholic liver cirrhosis is associated with a decline in serum afamin, perhaps due to impaired hepatic synthesis (18). Evidence evaluating a possible role of afamin as a marker of hepatic lipid accumulation and liver disease in a context of obesity and type 2 diabetes is not available.

The aim of this study was to investigate in detail afamin`s regulation in patients with obesity, prediabetes & newly diagnosed T2D and to evaluate effects of 3-month exercise intervention in middle-aged overweight-to-obese individuals on whole-body metabolism and serum afamin to determine the best clinical correlates of elevated serum afamin.

## 2 Materials and Methods

### 2.1 Design of Clinical Studies and Study Populations

Both the cross-sectional and exercise intervention study were conducted at the Institute of Experimental Endocrinology, Biomedical Research Center, Slovak Academy of Sciences in Bratislava, Slovakia; a detailed study protocol as well as the primary outcomes were published elsewhere (19). The studies were approved by the Ethics Committee of the University Hospital Bratislava and conform to the ethical guidelines of the 2000 Helsinki declaration. Subjects taking regular pharmacotherapy or food supplements, smokers, and those suffering from malignant, cardiovascular or other chronic disease other than newly diagnosed, yet-untreated T2D and hepatic steatosis were not eligible for the study. All participants provided witnessed written informed consent before entering the study.

### 2.2 Cross-Sectional Study (Study 1)

This was a single-center observational study with a cross-sectional design. Obese men with normal glucose tolerance and fasting glycemia (n=19) and patients with prediabetes (n=17) or untreated, newly diagnosed T2D (n=16) with a similar degree of obesity were compared to lean healthy controls (n=19, characteristics in **Table 1**). Clinical examinations (phenotyping) included anthropometric parameters (body weight/BMI, waist circumference), body composition (bioelectrical impedance, Omron BF511, Japan), whole-body insulin sensitivity (euglycemic hyperinsulinemic clamp). Subcutaneous abdominal adipose tissue biopsies were taken in order to measure adipocyte diameter. Glucose tolerance was assessed by oral glucose tolerance test (oGTT). Abdominal adipose tissue distribution was analyzed with magnetic resonance imaging (MRI) and hepatic lipid content with proton magnetic resonance spectroscopy (^1^H-MRS) (Symphony 1.5T spin echo; Siemens, Erlangen, Germany). Habitual ambulatory activity (step count, % of moderate and high intensity activity) was monitored with accelerometers (Lifecorder PLUS, Kenz, Japan) for at least 3 consecutive days and self-assessed physical activity score was obtained using validated questionnaire (20).

**Table 1:** Characteristics of the study population (Study 1).

	Lean (n = 19)	Obese (n = 19)	Prediabetes (n = 17)	T2D (n = 16)
**Age** [years]^#^	32.8 ± 6.7	37.1 ± 8.4	42.2 ± 10.0^*^	49.6 ± 8.6^*‡^
**BMI** [kg/m^2^]	22.3 ± 2.1	31.6 ± 2.7^*^	32.4 ± 2.5^*^	31.2 ± 3.9^*^
**Body fat** [%]^#^	16.9 ± 4.2	30.0 ± 3.8^*^	30.9 ± 3.0^*^	29.9 ± 5.4^*^
**Visceral AT** [cm^2^]^#^	84.1 ± 15.5	119.5 ± 34.0^*^	141.6.1 ± 45.0^*^	190.0 ± 42.9^*‡^
**Adipocyte diameter** [µm]	89.4 ± 12.6	120.0 ± 11.5^*^	121.0 ± 12.0^*^	118.8 ± 16.0^*^
**Fasting glucose** [mmol/l]	4.7 ± 0.5	4.8 ± 0.5	5.7 ± 0.8	8.9 ± 2.6^*‡†^
**2h glucose oGTT** [mmol/l]	5.5 ± 1.1	5.3 ± 1.5	7.7 ± 1.7^*‡^	13.6 ± 3.0^*‡†^
**Glucose AUC** [oGTT]	25.0 ± 3.4	26.7 ± 4.1	37.5 ± 7.4^*‡^	53.4 ± 5.8^*‡†^
**Fasting insulin** [μU/ml]^#^	5.31 ± 4.05	11.77 ± 11.16	14.89 ± 8.04^*^	13.34 ± 8.33^*^
**Insulin AUC** [oGTT]^#^	219.5 ± 83.1	324.2 ± 180.0	384.9 ± 169.2^*^	236.3 ± 170.7
**C-peptide** [mmol/l]^#^	1.28 ± 0.86	2.02 ± 1.17	2.76 ± 1.01^*^	2.91 ± 1.32^*^
**Triglycerides** [mmol/l]^#^	0.97 ± 0.35	1.24 ± 0.46	2.16 ± 1.01^*^	2.15 ± 1.26^*^
**Total cholesterol** [mmol/l]^#^	4.13 ± 0.81	4.35 ± 0.70	4.90 ± 1.09	5.22 ± 0.76^*‡^
**LDL cholesterol** ^#^	2.30 ± 0.67	2.58 ± 0.52	2.71 ± 0.85	2.98 ± 0.59^*^
**Atherogenic index** ^#^	2.04 ± 0.67	2.79 ± 0.54^*^	3.07 ± 0.62^*^	3.16 ± 0.61^*^
**hsCRP** [mg/l]^#^	0.66 ± 0.63	2.32 ± 2.08^*^	2.55 ± 1.76^*^	2.37 ± 1.37^*^
**Insulin effect**				
**M-value** [mg.kg^-1^.min^-1^]^#^	8.55 ± 2.70	4.75 ± 1.96^*^	3.21 ± 0.98^*^	3.06 ± 2.23^*^
**M-value/Ins** [mg.kg^-1^.min^-1^/μU.ml^-1^ of insulin]^#^	0.15 ± 0.06	0.08 ± 0.04^*^	0.05 ± 0.02^*^	0.06 ± 0.05^*^
**HOMA-IR** ^#^	1.13 ± 0.88	2.53 ± 2.43	3.79 ± 1.97^*^	5.29 ± 3.66^*^
**FFA-EHC [**mmol/l]^#^	0.12 ± 0.05	0.15 ± 0.04	0.18 ± 0.12	0.17 ± 0.10
**Liver parameters**				
**Hepatic lipid content** [% of water resonance, Log]	0.09 ± 0.27	0.45 ± 0.60	1.05 ± 0.52^*‡^	1.18 ± 0.52^*‡^
**Fatty liver index** ^#^	4.34 ± 4.23	39.84 ± 16.55^*^	62.65 ± 23.44^*^	57.70 ± 28.72^*^
**AST** [μkat/l]^#^	0.26 ± 0.04	0.36 ± 0.10^*^	0.34 ± 0.13	0.36 ± 0.20
**ALT** [μkat/l]^#^	0.15 ± 0.12	0.28 ± 0.12^*^	0.27 ± 0.15^*^	0.26 ± 0.12^*^
**GGT** [μkat/l]^#^	0.32 ± 0.23	0.58 ± 0.38^*^	0.84 ± 0.48^*^	0.74 ± 0.46^*^
**Physical activity**				
**Steps** [number/24h]^#^	9702 ± 3533	8158 ± 3035	8408 ± 5496	10385 ± 5790
**% of moderate and high intensity PA** ^#^	44.8 ± 13.4	40.1 ± 16.1	32.8 ± 14.9	25.0 ± 16.1^*‡^
**Leisure time index** ^#^	3.6 ± 0.5	2.7 ± 0.8^*^	2.7 ± 0.7^*^	2.8 ± 0.7^*^
**Sport index** ^#^	2.74 ± 0.82	2.30 ± 0.85	1.97 ± 0.68^*^	2.39 ± 0.85

Data are expressed as means ± SD. *significant as compared to lean controls, ^‡^patients with obesity, ^†^prediabetes. P < 0.05; 1-way ANOVA and Tukey`s post-hoc test were used to compare the groups; ^#^Parameters shown non-normal distribution in at least one group - nonparametric testing was applied (Kruskal-Wallis test with Dunn’s multiple comparison test).

ALT, alanine aminotransferase; AST, aspartate aminotransferase, AT, adipose tissue; AUC, area under the curve; BMI, body mass index; FFA-EHC, Free Fatty Acids measured in a steady state of the euglycemic hyperinsulinemic clamp/EHC; GGT, gamma-glutamyltransferase; HOMA-IR, HOMA insulin resistance index; hsCRP, high sensitivity C-reactive protein; M-value/Ins, insulin sensitivity normalized to steady state insulinemia, measured by EHC; oGTT, oral glucose tolerance test; PA, physical activity (assessed by accelerometers).

### 2.3 Exercise Intervention Study (Study 2)

Twenty-two sedentary overweight-to-obese non-diabetic middle-aged individuals (16/6 M/F) underwent an intensive supervised 12-week strength or aerobic training program (three sessions/week), as described in (19). While strength training included resistance machine and free-weight exercises of progressively increasing intensity for strengthening upper and lower body, the aerobic training consisted of aerobic dancing, running and spinning exercises at intensity of 70-85% of maximal heart rate (60 min/session).Volunteers were advised to maintain their dietary habits during the intervention. Complex clinical phenotyping, performed before and after training intervention, included assessments of BMI, waist circumference, glucose tolerance (oGTT), insulin resistance (HOMA-IR), body composition (Omron BF511, Japan). Fasting blood was collected for biochemical analyses (glucose, insulin, plasma lipids, liver enzymes). Volume and intensity of habitual ambulatory activity were objectively measured with accelerometers during the entire 3-month period of exercise intervention (Lifecorder PLUS, Kenz, Japan). Physical activity level was assessed by validated questionnaires (20). Visceral and subcutaneous abdominal adipose tissue content/distribution was evaluated by MRI and hepatic lipids by ^1^H-MRS (Symphony 1.5T spin echo; Siemens, Erlangen, Germany). Maximal oxygen consumption (VO_2_max) was determined as previously described in (19). Maximal isometric force was measured on a linear leg press dynamometer in a sitting position with a knee angle of 90° (21).

### 2.4 Biochemical Assays (Study 1 and 2)

Serum glucose concentration was measured using the glucose oxidase method (Beckman Coulter, Brae, CA, USA), insulin by IRMA (Immunotech, Marseille, France), total and HDL cholesterol, and triglycerides with kits from Roche (Germany), free fatty acids (FFA) and hsCRP were measured with assay kits from Randox (Crumlin, UK). The atherogenic index [log (triglyceride/HDL-cholesterol)] and LDL cholesterol concentrations (Friedewald equation) were calculated. Adiponectin was measured in fasting serum with ELISA (BioVendor, Brno, Czech Republic). C-peptide, AST, ALT and GGT were measured in a certified laboratory (Alpha Medical, Bratislava, Slovakia). Fatty liver index was calculated according to (22).

### 2.5 Oral Glucose Tolerance Test (Study 1 and 2)

After an overnight fast, an indwelling catheter was placed into an antecubital vein for blood sampling. Blood samples were drawn before (0 min) and 30, 60, 90, and 120 min after ingestion of 75g glucose and used to determine concentrations of plasma glucose, insulin, and C-peptide. Areas under the glycemic and insulinemic curves and HOMA-IR were calculated. Blood pressure was measured using Dinamap Vital Signs Monitor 845 XT (Critikon Inc., Tampa, FL, USA) after 30min rest.

### 2.6 Whole-Body Insulin Sensitivity (M-Value) (Study 1)

One-step frequently sampled hyperinsulinemic-euglycemic clamp was performed between 09.00h and 11.30h after an overnight fast. A primed (80 mU.m^-2^.min^−1^, first 10 min) continuous (40 mU.m^-2^.min^−1^) insulin (Actrapid^®^, Novo Nordisk, Bagsvaerd, Denmark) infusion was used to achieve hyperinsulinemia. Euglycemia (5 ± 0.25 mmol.l^-1^) was maintained by frequent (every 5 min) glucose measurements and appropriate rate adjustments of 20% glucose infusion. The whole-body insulin sensitivity (M-value) was calculated from the glucose infusion rate required to maintain euglycemia during steady state (90 min – 150 min of the clamp) and expressed as mmol of glucose per kilogram of body weight per minute. Insulin sensitivity index was calculated as M-value/insulinemia during steady state of EHC [19]. Adipose tissue insulin sensitivity was determined as the capacity to decrease circulating levels of free fatty acids during the steady state euglycemic hyperinsulinemia (FFA-EHC).

### 2.7 Abdominal Adiposity and Hepatic Lipid Content (Study 1 and 2)

Abdominal fat distribution was measured by MRI using GRE sequence, TR 134 ms, TE 2.38 ms, on a 1.5 T Magnetom Symphony MRI scanner (Siemens Healthcare, Erlangen, Germany). The visceral and subcutaneous abdominal adipose tissue content was evaluated as an average surface area of five consecutive slices (1cm distance) centered between the L4 and L5 vertebrae, segmentation was performed manually (Image J). Hepatic lipid content was determined by using (PRESS) sequence TR/TE/ACQ = 4000 ms/30 ms/4; VOI = 3 cm × 3 cm × 3 cm. The amount of hepatic lipids was expressed as percentage of water resonance. Spectra were processed by jMRUI 3.0 software.

### 2.8 Adipocyte Diameter (Study 1)

Subcutaneous adipose tissue samples were taken by needle biopsy from the abdominal (umbilical) region after an overnight fast. Adipocyte diameter was determined by histomorphometry of collagenase digestion-isolated adipocytes. Average diameter of at least 100 cells from each adipocyte suspension was calculated.

### 2.9 Quantification of Serum Afamin (Study 1 and 2)

Afamin concentrations were quantified in human serum in duplicates with a commercially available sandwich-type ELISA (BioVendor, Brno, Czech Republic), using two different specific monoclonal antibodies recognizing human afamin as modified from the previously described protocol (17). Recombinantly expressed and purified human afamin served as assay standard. According to the manufacturer’s protocol, within-run and run-to-run coefficients of variations were 3.6% and 3.4%, respectively, at a mean afamin concentration of 80 mg/l (17).

### 2.10 Statistical Analyses

Normality of data distribution was assessed with the Shapiro-Wilk test and log transformation of the data was used when appropriate. Differences between repeated measurements of the same group were analyzed with paired Student’s t-test. Nonparametric tests (Wilcoxon matched pairs signed rank test) were used where appropriate. Differences across more than two groups were evaluated with a 1-way ANOVA followed by Tukey post-hoc test. For non-parametric data, Kruskal-Wallis test with Dunn’s multiple comparison test was used. Differences between three or more repeated measurements of the same group were analyzed with 1-way repeated measures ANOVA. Associations between variables were analyzed with Spearman correlation analysis (if not indicated otherwise) and multiple linear regression analysis was employed. Statistical analysis was performed in JMP (version 4.0.4 academic; SAS Institute, USA) and GraphPad Prism 6 (GraphPad Software Inc.). Values are presented as mean ± SD. The significance level was set at p<0.05.

## 3 Results

### 3.1 Cross-Sectional Study (Study 1)

#### 3.1.1 Characteristics of the Study Population

Afamin was evaluated in a subpopulation of a larger clinical study (19), and population characteristics are given in **Table 1**. In brief, fasting and 2h-glycemia were used to diagnose prediabetes (increased fasting glucose or impaired glucose tolerance) and T2D. Pathophysiological markers of metabolic disease included the level of visceral and subcutaneous adiposity, adipocyte diameter, markers of chronic systemic inflammation (hsCRP), hepatic lipotoxicity (ALT, AST, GGT, hepatic lipids), atherogenic risk and whole-body insulin sensitivity (M-value). Patients with prediabetes and T2D displayed increased fasting insulin, C-peptide and triglycerides as well as insulin resistance (HOMA-IR) and visceral adiposity, when compared to lean healthy controls; and increased 2h glycemia and hepatic lipid content, when compared to lean and obese individuals without prediabetes. Hepatic lipid content was higher in subjects with IFG but not IGT, as compared to obese/metabolically healthy individuals (p<0.05). Patients with T2D had higher total cholesterol concentrations compared to lean and obese individuals and higher fasting, 2h-glycemia and area under the glycemic curve (oGTT) when compared to all other groups. Moreover, patients with T2D had lower leisure time index (Baecke questionnaire), when compared to lean healthy individuals and lower levels of moderate and high intensity habitual physical activity (intensity >3MET) as assessed by accelerometers, when compared to metabolically healthy lean or obese individuals (**Table 1**).

#### 3.1.2 Serum Afamin Concentrations in Obesity, Prediabetes and T2D

**Figure 1:**
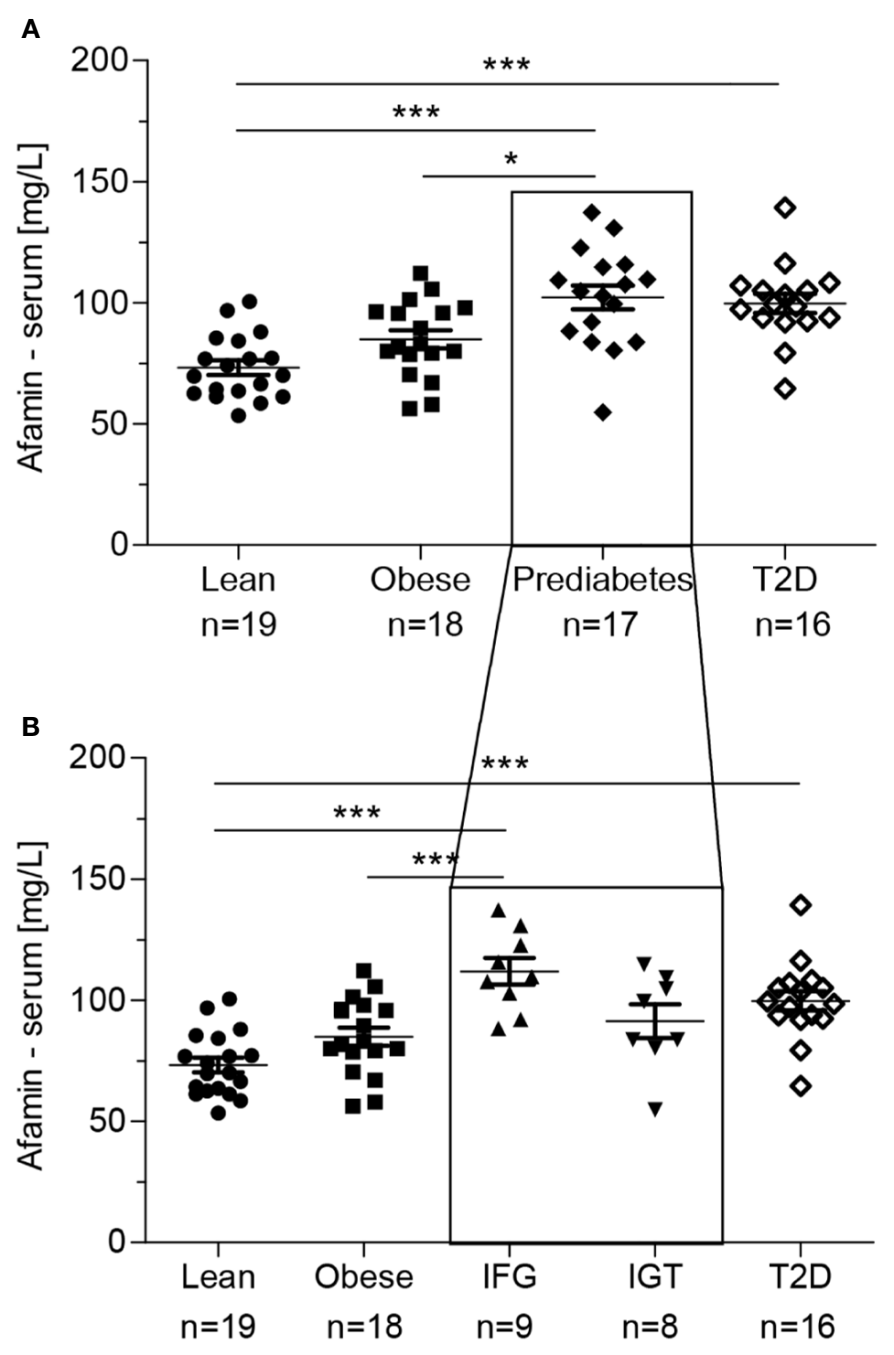
**(A)** Serum afamin concentrations in individuals with obesity, prediabetes and type 2 diabetes (T2D). **(B)** The group with prediabetes was divided into IFG (Increased Fasting Glucose) and IGT (Impaired Glucose Tolerance). Data are presented as mean ± SEM. *p < 0.05; ***p < 0.001; 1-way ANOVA followed by Tukey post-hoc test. 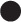 lean-healthy; ■ obese-healthy; ♦ obese – prediabetes; ▲ obese-increased fasting glucose (IFG); ▼ obese-impaired glucose tolerance (IGT); ⋄ obese - type 2 diabetes (T2D).

Serum afamin concentrations were increased in patients with prediabetes and T2D compared to lean healthy controls (p<0.001, **Figure 1A**), and in patients with prediabetes compared to individuals with obesity (p<0.05, **Figure 1A**). When adjusted for age, afamin remained significantly different between lean healthy controls and patients with prediabetes (p<0.001) and T2D (p< 0.05), however not between obese and prediabetic patients. These observations suggest that higher serum afamin is not related to obesity per se, but it seems to reflect disturbances in glucose metabolism and insulin action.

Moreover, patients with increased fasting glucose (IFG) but not those with isolated impaired glucose tolerance (IGT) had higher circulating afamin concentrations as compared to metabolically healthy lean (p<0.001) and obese (p<0.001) individuals (**Figure 1B**). The differences remained significant after adjusting afamin for age (with lean p<0.001; with obese p<0.05). It is important to note, that the highest measured serum afamin level (147.66 mg/l) in this cohort was detected in the obese highly insulin resistant (M value/Ins: 0.014 mg.kg^-1^.min^-1^/μU.ml^-1^, HOMA-IR: 11.51) and hyperinsulinemic (53.4 μU/ml) subject with normal fasting glucose (4.85 mmol/l) and glucose tolerance (2h glucose: 5.69 mmol/l). This individual was excluded from the analysis of serum afamin (above) as an outlier, but all the aforementioned outcomes were preserved independent on his exclusion, except for the difference between patients with obesity and prediabetes.

#### 3.1.3 Serum Afamin Correlates With Metabolic and Physical Activity Phenotypes

Afamin correlated with various metabolic phenotypes (**Table 2**). Positive correlations were found between afamin and determinants of obesity (BMI, body weight, % of body fat), abdominal adiposity (waist circumference, subcutaneous and visceral adiposity), subcutaneous adipocyte diameter (fat cell size), systemic markers of metabolic overload and metabolic disorders e.g. fasting triglycerides, insulin, C-peptide, glucose, HOMA-IR, atherogenic index, hsCRP, FFA, area under the glycemic, insulinemic and C-peptide curve (oGTT). On the other hand, afamin correlated negatively with whole-body insulin sensitivity (M-value/Ins), adipose tissue insulin sensitivity (magnitude of insulin-induced FFA suppression during euglycemic-hyperinsulinemic clamp), as well as with serum adiponectin and characteristics of habitual physical activity (steps per day, sport and leisure time indexes; **Table 2**). Most correlations of afamin with clinical parameters remained significant when adjusted for age (**Table 2**).

**Table 2:** Afamin correlates with parameters of obesity, insulin resistance & physical activity.

			Afamin adjusted for age		
	r	p	r	p	N
Age	0.366	<0.01			71
**Obesity**					
Atherogenic index	0.589	<0.0001	0.419	<0.001	69
BMI	0.529	<0.0001	0.419	<0.001	71
Body fat %	0.526	<0.0001	0.428	<0.001	68
Fat cell size	0.519	<0.0001	0.392	<0.01	67
Waist circumference	0.513	<0.0001	0.409	<0.001	69
Fasting triglycerides	0.485	<0.0001	0.396	<0.001	70
Body weight	0.458	<0.0001	0.379	<0.01	71
hsCRP	0.444	<0.001	0.377	<0.01	67
Visceral AT	0.431	<0.001	0.251	<0.05	64
Subcutaneous AT	0.401	<0.01	0.305	<0.05	64
Fasting free fatty acids	0.312	<0.01	0.280	<0.05	70
Systolic blood pressure	0.206	>0.05	0.193	>0.05	58
Fasting cholesterol	0.214	>0.05	0.108	>0.05	70
LDL cholesterol	0.163	>0.05	0.053	>0.05	69
**Liver parameters**					
Hepatic lipid content	0.721	<0.0001	0.595	<0.0001	60
GGT	0.656	<0.0001	0.567	<0.0001	70
Fatty liver index	0.655	<0.0001	0.536	<0.0001	68
ALT	0.528	<0.0001	0.496	<0.0001	70
AST	0.330	<0.01	0.320	<0.01	69
**Diabetes**					
HOMA-IR	0.674	<0.0001	0.600	<0.0001	70
Fasting Insulin	0.646	<0.0001	0.616	<0.0001	70
M-value/Ins*	-0.632	<0.0001	-0.560	<0.0001	70
Fasting C-peptide	0.614	<0.0001	0.516	<0.0001	69
Insulinemia AUC (oGTT)	0.593	<0.0001	0.599	<0.0001	61
Serum adiponectin	-0.536	<0.0001	-0.478	<0.001	48
Fasting glycemia	0.473	<0.0001	0.291	<0.05	71
C-peptide AUC (oGTT)	0.417	<0.001	0.389	<0.01	61
Glycemia AUC (oGTT)	0.396	<0.01	0.273	<0.05	62
FFA-EHC**	0.346	<0.01	0.365	<0.01	69
**Physical activity**					
Steps number/24h	-0.268	<0.05	-0.327	<0.05	60
Sport index	-0.267	<0.05	-0.229	>0.05	71
Leisure-time index	-0.251	<0.05	-0.229	>0.05	71

ALT, alanine aminotransferase; AST, aspartate aminotransferase; AT, adipose tissue; AUC, area under the curve; BMI, body mass index; FFA-EHC, Free Fatty Acids measured in a steady state of the euglycemic hyperinsulinemic clamp/EHC; GGT, gamma-glutamyltransferase; HOMA-IR, HOMA insulin resistance index; hsCRP, high sensitivity C-reactive protein; M-value/Ins, insulin sensitivity normalized to steady state insulinemia, measured by EHC; Spearman correlation. *whole body insulin sensitivity, **adipose tissue-specific insulin sensitivity.

#### 3.1.4 Relationship Between Serum Afamin Concentrations and Liver Parameters

**Figure 2:**
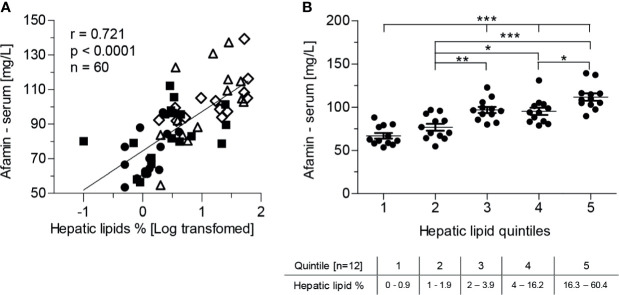
Serum afamin concentrations and hepatic lipid content, measured by ^1^H-MR spectroscopy (study 1). **(A)** Serum afamin correlates positively with hepatic lipids (Pearson linear correlation) **(B)** Stratification of the study population into quintiles according to hepatic lipid content. Data are presented as mean ± SEM. *p < 0.05; **p < 0.01; ***p < 0.001; 1-way ANOVA followed by Tukey post-hoc test; 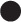 lean-healthy; ■ obese-healthy; Δ obese -prediabetes, 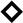 obese - type 2 diabetes.

The main source of circulating afamin is the liver. Therefore, we analyzed relationships of serum afamin concentrations with liver metabolic and functional parameters. Afamin was positively correlated with markers of liver damage such as AST, ALT and GGT and with the fatty liver index (**Table 2**). Furthermore, there was a strong positive correlation between afamin and hepatic lipid content (**Figure 2A**). All these associations remained significant when adjusted for age (**Table 2**, hepatic lipids: r=0.595, p<0.0001, n=60). Stratification of the study population into quintiles according to the hepatic lipid content revealed that a relatively small increase in the hepatic lipids was paralleled by a significant increase in serum afamin (**Figure 2B**).

Multiple regression analysis identified fasting insulinemia (p=0.0004) and hepatic lipid content (p=0.0001) as the major determinants of serum afamin, explaining >63% of its variability in a model comprising age, BMI, fasting insulinemia and hepatic lipid content (r^2^=0.635, p<0.0001) (**Table 3**). Obesity per se seemed not to be associated with elevated afamin, since neither BMI (p=0.764) nor body weight (p=0.803), waist circumference (p=0.908), % of body fat (p=0.740), visceral (p=0.693), subcutaneous adipose tissue (p=0.516), adipocyte diameter (p=0.903) had a significant contribution in a regression model consisting of age, hepatic lipids, and fasting insulin. When other hepatic parameters such as GGT (r^2^=0.561; p=0.0004) or ALT (r^2^=0.516; p=0.014) replaced liver lipid content in the regression model, they both were significant predictors of increased afamin together with fasting insulinemia, albeit the most pronounced effect was found with hepatic lipid content. Replacing fasting insulinemia with other parameters of insulin-resistance/sensitivity e.g. (M-value/Insulin r^2^=0.571; p=0.031) or area under the insulinemic curve (oGTT) (r^2^=0.599; p=0.003) revealed, that both could explain some of the variability in serum afamin concentration.

**Table 3:** Regression analysis showing independent predictors of serum afamin concentrations (using multiple linear regression analysis).

Model	Unstandardized coefficients		t	Sig.	95.0% Confidence interval for B	
	B	Std.Error			Lower Bound	Upper Bound
R^2^ = 0.635						
Constant	65.858	12.893	5.108	<0.0001	40.008	91.707
Age [years]	0.127	0.202	0.626	0.534	-0.279	0.532
BMI [kg/m^2^]	-0.148	0.488	-0.302	0.764	-1.127	0.832
Fasting insulin [μU.ml^-1^]	1.177	0.309	3.803	0.0004	0.557	1.797
Hepatic lipids [% of water resonance, Log]	15.247	3.731	4.086	0.0001	7.766	22.729

Serum concentrations of afamin can predict the accumulation of hepatic lipids to the same extent (r^2^=0.52, p<0.001) as the clinically relevant fatty liver index, calculated from serum lipids, GGT, BMI and waist circumference (r^2^=0.50; p<0.001).

#### 3.1.5 Afamin Is Not Markedly Regulated by Oral Glucose Load

We examined afamin concentrations during the frequently sampled 2h oGTT, which was characterized by elevated glycemia and insulinemia (**Supplementary Figures 1A, B**). We observed a slight but significant decrease of afamin concentrations within the first 60min of the oGTT in patients with prediabetes and T2D (**Supplementary Figure 1C**). Apart from this small change, afamin concentrations during oGTT were rather stable across the groups, which supports previous findings that afamin is a good and stable marker, minimally affected by the prandial state.

#### 3.1.6 Exercise Training and Serum Afamin (Study 2)

We analyzed the effects of 3-month aerobic and strength training intervention to modulate circulating afamin concentrations. Population comprised of sedentary middle-aged men and women (37 ± 5.4 yrs., n=22; M/F, 16/6) with overweight (n=9) or obesity (n=13) (BMI: 31.2 ± 3.2 kg/m^2^), with average baseline afamin levels of 100.97 ± 20.96 mg/l. 3 subjects had impaired glucose tolerance, 1 increased fasting glucose and 1 had both.

The 3-month supervised exercise intervention induced favorable changes in body composition without significant effects on body weight and BMI. It reduced waist circumference (p<0.01; n=18), % of body fat (p<0.05; n=22) and the volume of visceral adipose tissue (p<0.01; n=17), while increasing lean body mass (p<0.05; n=22), level of aerobic cardiorespiratory fitness VO_2_max (p<0.05; n=18) and muscle strength (p<0.01; n=19). Effect on hepatic lipids was not significant (p=0.268).

**Figure 3:**
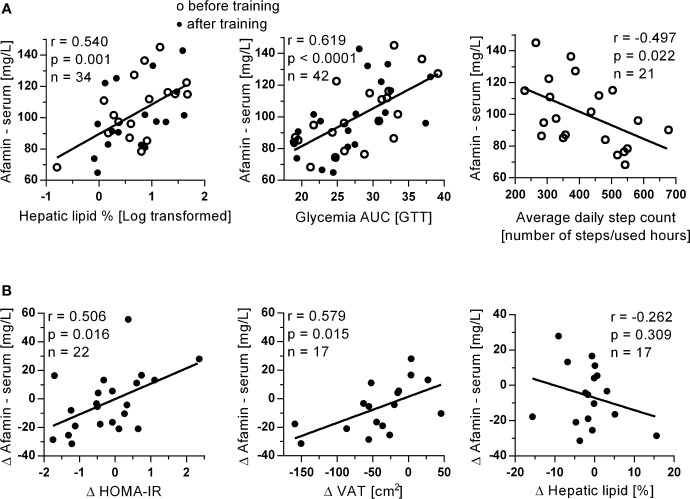
Associations of **(A)** serum afamin concentrations with hepatic lipids, glycemia AUC and average daily steps count determined during the entire 12-week exercise intervention in study 2. Associations of **(B)** changes in serum afamin concentrations and changes in clinical parameters, induced by the 3-month exercise training intervention. Δ: value after training intervention (trained) – value before training (untrained); AUC, area under the curve; HOMA-IR, index of insulin resistance; oGTT, oral glucose tolerance test; VAT, visceral adipose tissue (assessed by MRI); Pearson linear correlation.

The 3-month exercise intervention had no significant effect on serum afamin (p=0.636, n=22), however, afamin was also in this cohort associated with body weight (r=0.372; p=0.013), lean body mass (r=0.364; p=0.015), fasting glycemia (r=0.454; p=0.020), HOMA-IR (r=0.343; p=0.023), fasting triglyceridemia (r=0.393; p=0.008) and abdominal visceral adiposity (r=0.500; p=0.002). Similar to study 1, strong positive and age-independent correlations were found between afamin and hepatic lipid content and with the area under the glycemic curve (oGTT), while negative correlation was found between afamin and habitual physical activity (average daily step count measured during the entire 12-week training period (**Figure 3A**).

More importantly, training-induced improvements in insulin resistance (fasting insulinemia: r=0.457; p=0.033; n=22, HOMA-IR) and visceral adiposity were positively associated with training-induced changes in circulating afamin concentration (**Figure 3B**). These associations were also independent on age (p<0.05). There was no correlation between training-induced changes in serum afamin concentrations and training-induced changes in hepatic liver content (**Figure 3B**).

## 4 Discussion

The exact physiological role of afamin in humans has not yet been elucidated. However, several large human association studies and a small pilot study in transgenic mice suggested interrelations between circulating afamin and the development/progression of various components of metabolic syndrome and diabetes (8–16, 23–25).

In our study, we measured afamin concentrations in a cohort of metabolically well-characterized men at different stages of metabolic disease development. We showed that serum afamin concentration was increased in individuals with prediabetes and newly diagnosed yet untreated T2D and that it positively correlated with many obesity and diabetes-related phenotypes e.g. body weight, BMI, waist circumference, % of body fat, subcutaneous and visceral adiposity, fasting glycemia, insulinemia, triglyceridemia, free fatty acids, atherogenic and insulin resistance (HOMA-IR) index and negatively with whole-body insulin sensitivity as assessed by EHC. Serum afamin also negatively correlated with adiponectin and with the level of habitual physical activity. Our findings complement those published by others reporting pronounced positive associations with waist circumference, BMI, fasting triglycerides, glycemia, insulinemia and HOMA-IR (8, 9, 16). In contrast with other reports, we did not find correlations of afamin with total and LDL cholesterol (8, 16), while Seeber et al., failed to find associations between afamin, waist circumference and fasting glycemia when examining patients with polycystic ovary syndrome and healthy control females.

In order to determine predictive power of afamin for the early stage metabolic and hepatic disease, multivariate linear regression analyses have been performed. Seeber at al. identified fasting triglyceridemia as an age- and obesity-independent predictor of circulating afamin (16). In our study, hepatic lipid content and fasting insulinemia were the strongest determinants of serum afamin. This is in line with the studies examining afamin in patients with polycystic ovary syndrome, where insulinemia and parameters of insulin resistance were identified as the best determinants of elevated circulating afamin (15, 16). The association between afamin and hepatic lipids was not explored, since the previous large epidemiological studies aimed at exploring afamin as an early marker of metabolic syndrome and T2D (8–10) did not measure hepatic lipid content.

The expression of the afamin gene is activated in the liver at birth and continues to be expressed at high levels in adults (2) and regulated by concerted action of hepatocyte nuclear factors 1a and 1b (26). Although liver is the main source of circulating afamin, there are no functional studies on afamin using human or animal liver cell models. Several publications have reported decreased afamin blood concentrations associated with severe alcoholic liver cirrhosis (18) and with liver cancer (27, 28). In our study, we found a strong positive association between serum afamin and hepatic lipid content, which we observed in two independent study populations. Importantly, hepatic lipid content was the strongest predictor of serum afamin in a model comprising age, BMI and parameters of obesity and insulin resistance. This is in line with recent proteomic studies demonstrating afamin as one of several blood markers for non-alcoholic fatty liver disease (NAFLD) examined in children (29) and in adults (30). Afamin has also been higher in T2D patients with NAFLD than in those without NAFLD (31), suggesting that higher afamin concentrations are more related to liver disease than to diabetes per se. Pathogenesis of NAFLD is closely related to systemic insulin resistance and increased flux of FFA and glycerol into the liver (32). NAFLD is a common hepatic manifestation of metabolic syndrome, which is linked to the hepatic insulin resistance and increased fasting glucose (33), with a weaker relationship to impaired glucose tolerance (34). In line with this, we observed the highest afamin concentrations in patients with increased fasting glucose, which was paralleled by the increase of hepatic lipids in this group.

Afamin has also been positively correlated with fatty liver index and circulatory markers of hepatic function e.g. ALT, AST, GGT, indicating that afamin could, to some extent, reflect liver functional capacity (reserve). Liver plays a crucial role in the patho/physiology of glucose metabolism. Hepatic glucose production is almost the exclusive source of circulating glucose in the fasting state (35). Hepatic insulin resistance is therefore the major determinant of fasting hyperglycemia manifested as increased fasting glucose (IFG) (36), which is in line with our observations of the highest circulating afamin concentrations in individuals with increased fasting glucose. Multiple regression analysis identified fasting insulinemia and whole-body insulin sensitivity as determinants of serum afamin. This and a strong relationship of serum afamin with HOMA-IR and fasting glycemia allows us to speculate that afamin is strongly associated with hepatic insulin resistance.

We observed that apart from the slight but significant decrease within the first 60 min of oral glucose tolerance test, which was specific for patients with prediabetes and T2D, afamin is not markedly regulated by acute oral glucose load. With this we confirm the previous observation by others (17) and state that larger cohorts with prediabetes and T2D could be required to confirm this observation.

Regular exercise is a powerful physiological stimulus with the capacity to improve metabolic health by reducing hyperlipidemia, hyperglycemia, accumulation of hepatic lipids and lowering the chronic subclinical inflammation (37, 38). The 3-month supervised exercise intervention in a cohort of middle-aged overweight individuals lowered waist circumference, whole-body and visceral adiposity, while increasing lean body mass, aerobic fitness and muscle strength. The 3-month exercise intervention was not effective in reducing hepatic lipids, fasting insulinemia, glycemia or serum afamin however, exercise training-induced changes in fasting insulinemia, HOMA-IR and visceral adiposity correlated positively with intervention-induced changes in serum afamin. These results support the hypothesis that afamin could serve as an early marker of the prodromal changes in lipid/carbohydrate metabolism leading to insulin resistance, which could be modulated by regular exercise.

Based on large epidemiological studies (Bruneck, SAPHIR, KORA F4), afamin has been proposed as a clinically relevant early marker of metabolic syndrome, T2D (8, 9), gestational diabetes and pre-eclampsia (10–12, 14, 25). Here we provide evidence that complements previous reports (29, 30) and clearly indicates that afamin could serve as a marker of increased hepatic lipid content, which is tightly linked to metabolic disease development. Moreover, hepatic lipids and fasting insulinemia were identified as the most important determinants of systemic afamin concentration. While 3-month intensive supervised exercise intervention was not effective in modulating systemic afamin concentrations, exercise-induced changes of HOMA-IR, insulinemia and visceral adiposity were correlated with changes in circulating afamin concentrations.

## Data Availability Statement

The original contributions presented in the study are included in the article/**Supplementary Material**. Further inquiries can be directed to the corresponding authors.

## Ethics Statement

The studies involving human participants were reviewed and approved by Ethics Committee of the University Hospital Bratislava. The patients/participants provided their written informed consent to participate in this study.

## Author Contributions

TK: data generation, analysis and interpretation, drafting the manuscript. ZK, MB: data generation and analysis. EZ: training program conception, data analysis. VB: ^1^H-MRS and MRI data acquisition. MK: data generation. DG, JP: data interpretation, critical reading of the manuscript. JU, BU, HD: afamin study conception and design, data generation, analysis and interpretation, editing and critical reading/revising of the manuscript. Experiments were carried out at the Institute of Experimental Endocrinology, Biomedical Research Center Slovak Academy of Sciences in Bratislava. Circulating afamin measurements were performed by HD - Institute of Genetic Epidemiology, Department of Genetics and Pharmacology, Medical University of Innsbruck, Austria. All authors contributed to the article and approved the submitted version.

## Funding

This work has been supported by EFSD & Lilly research fellowship 2010-2013, Grant Agency of the Slovak Academy of Sciences [VEGA 2/0107/18; VEGA 2/0164/20], FP-EC-LipidomicNET #202272, European Regional Development Fund - OP Integrated Infrastructure [ITMS: 313011V344] and the Austrian Research Grant [FWF, P19969-B11].

## Conflict of Interest

Author VB was employed by the company Pro Diagnostic Group.

The remaining authors declare that the research was conducted in the absence of any commercial or financial relationships that could be construed as a potential conflict of interest.

## Publisher’s Note

All claims expressed in this article are solely those of the authors and do not necessarily represent those of their affiliated organizations, or those of the publisher, the editors and the reviewers. Any product that may be evaluated in this article, or claim that may be made by its manufacturer, is not guaranteed or endorsed by the publisher.
