# Supplementary material for: Serum Afamin a Novel Marker of Increased Hepatic Lipid Content
Source: Front Endocrinol (Lausanne). 2021 Sep 16;12:670425. doi: 10.3389/fendo.2021.670425 (PMC8481912; doi:10.3389/fendo.2021.670425)
Supplement: Supplementary file 1 [file DataSheet_1.pdf]

Supplementary Material

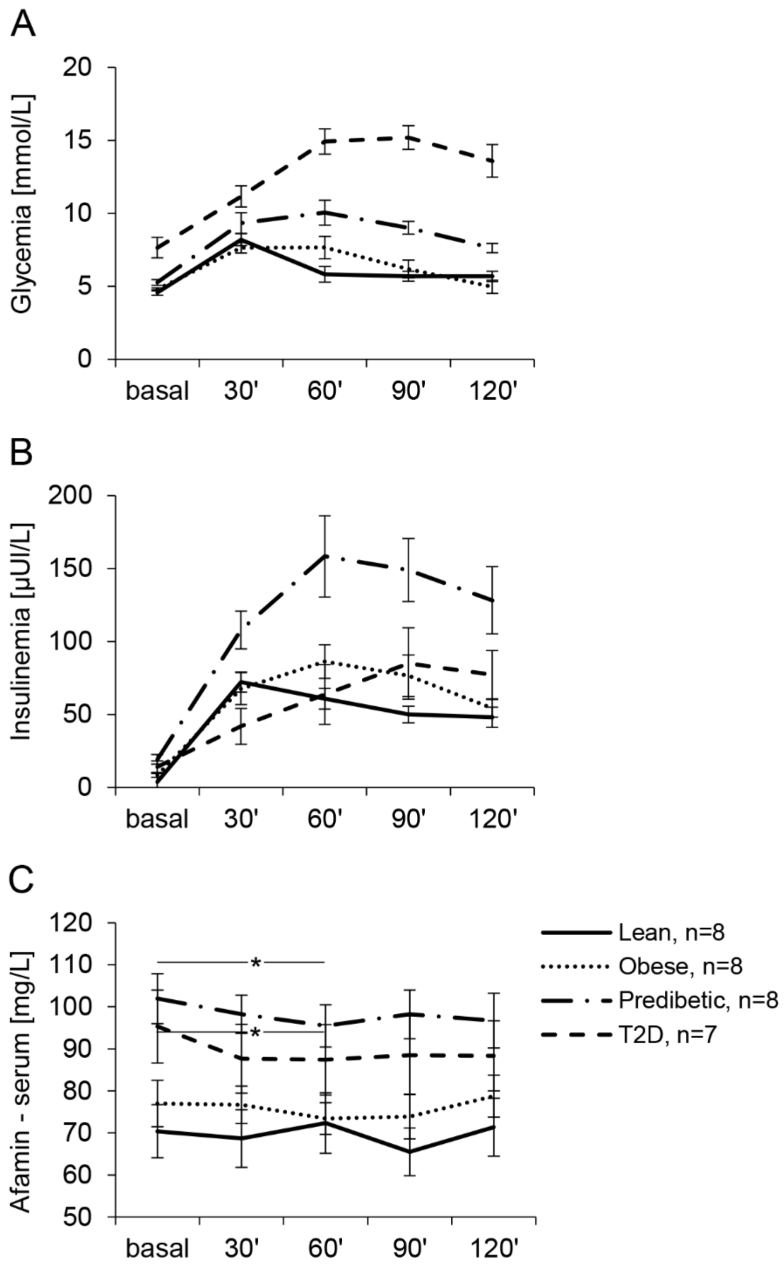

Supplementary Figure 1. (A) Glycemia, **(B)** insulinemia and **(C)** serum afamin concentrations during oGTT. Data are presented as mean ± SEM. * p<0.05; One-way repeated measures ANOVA (Turkey multiple comparison test).
